# Perceived benefits and barriers to exercise and associated factors among Zimbabwean undergraduate students: a cross-sectional study

**DOI:** 10.3389/fspor.2024.1205914

**Published:** 2024-08-07

**Authors:** Beatrice K. Shava, Blessed Vhudzijena, Tariro Kupenga-Maposa, Thelma Musingwini, Tanaka Samudzi, Sidney Muchemwa, Dixon Chibanda, Jermaine M. Dambi

**Affiliations:** ^1^ Rehabilitation Sciences Unit—Faculty of Medicine and Health Sciences, University of Zimbabwe, Harare, Zimbabwe; ^2^ Mental Health Unit—Faculty of Medicine and Health Sciences, University of Zimbabwe, Harare, Zimbabwe

**Keywords:** common mental disorders, health-related quality of life, undergraduate students, non-communicable diseases, barriers and benefits to exercise

## Abstract

**Background:**

Despite the well-documented benefits of regular physical activity (PA), many university students are physically inactive. Personal, socio-economic, and environmental factors predict PA engagement behaviours in university students. There is a need to understand context-specific perceived barriers and benefits to exercise engagement and physical activity levels amongst university students from low-income settings. This study primarily evaluated the barriers and facilitators to PA engagement in Zimbabwean undergraduate students. We also assessed the correlates of perceived barriers and benefits to PA engagement, risk of common mental disorders (CMDs) and health-related quality of life (HRQoL).

**Methods:**

We used a cross-sectional study to recruit 465 university undergraduate students. The Exercise Benefits and Barriers Scale, International Physical Activity Questionnaire (IPAQ), Shona Symptoms Questionnaire (SSQ-8) and EuroQol 5 Dimension (EQ5D-5l) were used to measure barriers and facilitators, physical activity level, risk of depression and anxiety and HRQoL, respectively. Data were analysed through descriptive statistics and logistic regression.

**Results and conclusion:**

Most participants were male (58.5%) with a mean age of 21.7 (SD 1.6) years. Majority of the participants were first year students (37.2%), consumed alcohol (66.5%), did not smoke (88.2%) and had a normal BMI (64.7%). The prevalence of low PA levels was 17.4%, with 33.5% of students at risk of CMDs. The most perceived benefits were in the physical performance (e.g., exercise improves my level of physical fitness) and life enhancement (e.g., exercise improves my self-concept) domains, while the most perceived barriers were lack of exercise infrastructure (e.g., exercise facilities do not have convenient schedules) and physical exertion (e.g., exercise tires me). Food insecurity (AOR 2.51: 95% CI 1.62–3.88) and the risk of CMDs (AOR 0.49: 95% CI 0.32–0.76) were associated with increased odds of experiencing barriers to exercise. Not using substances (AOR = 2.14: 95% CI 1.11–4.14) and a higher self-rated HRQoL (AOR 24.34: 95% CI 1.77–335.13) were associated with increased odds of a high perception of exercise benefits. Improving access to community and on-campus exercise facilities and campus-wide health promotional interventions is necessary to enhance PA amongst university students.

## Introduction

Physical activity (PA) is any body movement that raises energy expenditure above the resting metabolic rate through muscle contraction (1). Globally, non-communicable diseases (NCDs) such as cancer, cardiovascular diseases, diabetes, and common mental disorders (CMDs) like depression and anxiety are endemic (2, 3). Also, NCDs are increasingly becoming the leading cause of mortality in sub-Saharan Africa, accounting for 37% of the mortality burden (4). The burden is eve n greater in low-income countries like Zimbabwe, where the NCD-related mortality is 39% (5). Further, the magnitude of mental disorders in Zimbabwe as is globally, is growing exponentially, with anxiety and depressive disorders affecting 2.8% and 4.0% of the Zimbabwean population, respectively (6). Unfortunately, a massive mental healthcare treatment gap (discrepancy between people in need of care against available care resources) exists globally, with low-resourced countries disproportionally affected (7). Given the high mental healthcare treatment gap in low-income countries, it is essential to explore multiple treatment strategies, including physical-activity interventions. The numerous health benefits of regular PA engagement include the prevention and management of NCDs is uncontested (8, 9). Unfortunately, PA is slowly being replaced by a more sedentary lifestyle in the modernised population. For instance, about 27.5% of the adult global population (10) are sufficiently physically active. Further, 23% and 31% of Zimbabwean males and females are physically active compared to 22.1% of the African region’s adult population (11, 12). University students are no exception; for example, the estimated prevalence of physical inactivity in European and Sub-Saharan African university students is approximately in the ranges of 60%–91% and 37.1–62.5%, respectively (13–16). Low PA levels in young adults are concerning as PA engagement behaviours persist throughout the lifespan. For example, physically inactive people in adolescence and young adulthood are unlikely to achieve ideal PA levels in adulthood (17). Thus, it is vital to intervene early and promote healthy lifestyles anchored on increasing PA levels among adolescents and young adults, particularly university students.

The Health Belief Model (HBM) is one of the leading models for understanding health behaviours, and designing and evaluation of interventions to increase positive health behaviours (18, 19). The HBM model posits that health-related behaviours are influenced by perceived; barriers, benefits, severity, self-efficacy, and susceptibility (20). For example, if individuals perceive a negative health outcome to be severe, perceive themselves to be susceptible to it, perceive the benefits of behaviours that reduce the likelihood of that outcome to be high, and perceive the barriers to adopting those behaviours to be low, then the individual is likely to engage in positive health-seeking behaviour(s) (21). Given the high burden of physical inactivity, it is essential to apply the HBM as a conceptual framework to understand the multiple factors that may influence the rate of physical activity engagement among university students. For example, past studies have demonstrated that internal factors (e.g., perceived barriers and benefits) and external/environmental factors (e.g., availability of exercise facilities) predict PA engagement behaviours and patterns among university students and other populations (22, 23). Commonly perceived benefits to exercise among university students include; enhanced vitality, social interaction, recreation, and better sleep quality (14, 17, 24, 25). Inversely, ubiquitous perceived barriers to exercise engagement include negative experiences at school during physical education, personal factors (e.g., motivation, self-consciousness about appearance), lack of social support, time constraints, physical exertion and lack of safe spaces to engage in physical activity (17, 25–27). Although there is a fair understanding of the common benefits and barriers to exercise engagement among university students, the variations in settings, behaviours and cultural norms warrant contextualised epidemiological data. Further, most of the information has been from high-income countries and may have limited applicability in low-income countries due to context differences (28). Also, a granular understanding of the context-specific perceived benefits to exercise is crucial to understanding and developing bespoke interventions targeting university students (29).

As the global burden of CMDs continues to rise, there is a great propensity to explore alternative and complementary non-pharmacological and transdiagnostic therapeutic options for managing CMDs (9). Physical activity is widely recognised as a valid treatment for anxiety and depression (8, 30, 31). A meta-analysis of prospective cohort studies revealed that people with high self-reported PA (vs. low PA) were at reduced odds of developing anxiety (adjusted odds ratio = 0.748 95%CI = 0.629, 0.889; *p*-value = 0.001) and depression (adjusted odds ratio = 0.83, 95%CI = 0.79, 0.88; *p* < 0.0001) (30, 32). Further, a systematic review to investigate the feasibility and efficacy of exercise therapy for patients with depression found that moderate-intensity exercise reduces depressive symptoms, with higher-dose exercise associated with greater overall functioning and well-being (33). For university students, greater perceived benefits of exercise are associated with greater physical engagement and higher physical activity levels, positively affecting mental health and health-related quality of life (HRQoL) (14). Several studies have shown HRQoL gains attributed to physical activity across different populations (34–36). However, there is a paucity of published evidence regarding physical activity levels and factors affecting exercise engagement among university students in the Sub-Saharan region. Of the few studies available, Kgokong and Parker (14) conducted a cross-sectional study among South African physiotherapy students, *N* = 296 to describe the levels of PA and benefits and barriers to exercise for undergraduate physiotherapy students attending university in the Western Cape of South Africa. This study suggests that undergraduate physiotherapy students in the Western Cape universities do not engage in adequate PA. In this group of students, benefits associated with high PA related to physical performance and barriers associated with low levels of PA related to physical exertion. However, the study only recruited physiotherapy students, who are more likely to be knowledgeable about exercise benefits and barriers, thus creating selection bias. It is essential to understand exercise behaviours across all healthcare programs. Also, participants were drawn from one province, a threat to the study’s internal and external validity. Further, South Africa is an upper-middle income country, and comparability of the study to other Sub-Saharan countries could be limited. Given the research gaps, and need for contextualised data, this paper primarily sought to investigate the barriers and facilitators to physical activity engagement in Zimbabwean undergraduate students. We also sought to determine correlates of perceived barriers and benefits to; physical activity engagement, mental health disorders and perceived HRQoL. Results from this study can aid in developing bespoke and contextual solutions to promoting physical activity to improve health and well-being among university students from low-income countries.

## Materials and methods

### Study design

#### Cross-sectional study

##### Setting

The study was conducted at the University of Zimbabwe (UZ) in Harare, Zimbabwe, between April and June 2022. The UZ is the largest tertiary institution in Zimbabwe, with about 21,000 students distributed across ten faculties.

### Participants

All full-time, registered and freely consenting undergraduate students from the ten faculties were eligible for recruitment into the study. Participants were approached in their respective lecture rooms. The researchers briefly explained the purpose of the study to the students and offered interested students the consent form and a more detailed explanation of the study procedure. All willing participants were consecutively enrolled into the study after signing the consent form.

### Sampling and sample size calculation

In an almost similar South African study, 38% (*p*_0_) of physiotherapy students reported experiencing barriers to physical activity (14). We expected Zimbabwean university students to face more significant barriers to PA engagement (*p*_1 _= 0.45) due to the ongoing socio-economic challenges; for example, inequitable access to on-campus gym facilities. At least 388 participants were required at a 95% confidence interval and an 80% goal power. The sample size was estimated using STATISTICA. Participants were consecutively recruited into the study.

### Instrumentation

#### Sociodemographic questionnaire

This questionnaire collected data on these variables: age, presence of a chronic condition, alcohol intake, substance or drug use, smoking status, the experience of adverse events and socio-economic status (SES) measured proxy through perceived financial and food security levels.

#### The exercise barriers and benefits scale (EBBS)

The EBBS is a 43-item questionnaire to determine the perceived barriers and benefits to exercise engagement and is scored on a four-point Likert scale. Responses range from four (strongly agree) to one (strongly disagree). The benefit component comprises 29 items categorised into five subscales, i.e., life enhancement (e.g., exercise improves the quality of my work), physical performance (e.g., exercising increases my level of physical fitness), psychological outlook (e.g., exercise decreases feelings of stress and tension), social interaction (e.g., exercising is a good way for me to meet new people), and preventive health (e.g., I will live longer if I exercise). The barrier component includes 14 items categorised into four subscales, i.e., exercise milieu/infrastructure (e.g., there are too few places for me to exercise), time expenditure (e.g., exercising takes too much of my time), physical exertion (e.g., exercise tires me), and family discouragement (e.g., my family members do not encourage me to exercise). The cumulative scores on the benefits and barriers are 29–116 and 14–56, respectively. Higher scores denote a higher perception of benefits and barriers (17, 37, 38). The EBBS has high internal consistency, yielding Cronbach's alpha reliability coefficients of 0.95 and 0.86 for the benefits and barriers scales, respectively (37). The EBBs also has strong longitudinal reliability, yielding test-retest reliability of *α *= .89 and *α *= .77 for the benefits and barriers subscales, respectively (37).

#### The shona symptoms questionnaire (SSQ-8)

Developed in Zimbabwe, the SSQ-8 is a diagnostically-robust CMDs screener to detect depression and anxiety The questionnaire has two responses, i.e., yes/no, with scores ranging from 0 to 8. Scores ≥6 indicate the risk of CMDs (39). The locally generated psychometric properties show good consistency (Cronbach's alpha = 0.68), sensitivity (87%), specificity (70%) and positive predictive value of 82% with the stated cut-point (39).

#### The EQ5D–5l

The EQ-5D 5l assesses HRQoL in five dimensions, i.e., mobility, self-care, usual activities, pain/discomfort, and anxiety/depression. Severity is measured on a five-point Likert Scale ranging from 1 = not at to 5 = all the time. The EQ-5D 5l also includes a visual analogue scale, ranging from 0 (worst health imaginable) to 100 (best health imaginable) (40, 41). Responses from the five domains can be converted to a single summary score (known as multi-attribute utility) where death and total health are represented by 0.00 and 1.00, respectively. It is a generic measure with excellent psychometric properties; normative scores for the Zimbabwean population are available (40). The EQ-5D is among the most widely used instrument internationally for the evaluation of HRQoL and known psychometric performance, i.e., it has excellent reliability, validity and responsiveness across a range of populations (41).

#### International physical activity questionnaires (IPAQ)

The IPAQ is a validated self-administered international instrument used to measure data on health-related physical activity over a seven-day recall period. The IPAQ-short provides analysis algorithms for the total volume and number of days to assess PA. It classifies PA into three levels, i.e., low, moderate and high [IPAQ (42)]. The IPAQ-SF is well applied outcome measure with robust psychometric performance (43).

#### Body mass index (BMI)

Participants were measured for their weight using a digital scale which measured to the nearest 0.1 kg and height with the participant in an anatomical position to the nearest 0.1 cm. BMI was calculated for the individual participants from the raw data collected.

### Procedure/ethical considerations

Permission and ethical approval to conduct the study were granted by the UZ dean of students, the Joint University of Zimbabwe, and the Parirenyatwa Hospital Research Ethics Committee (JREC/148/2022). All participants signed a consent form before enrolment into this study.

### Data analysis

Descriptive statistics (e.g., frequencies, means) were used to describe participants’ characteristics and standardised outcomes. Logistic regression evaluated factors associated with perceived barriers and exercise benefits. First, crude odds ratios were calculated. All variables yielding values *p* ≤ .10 were fed into the multivariate binary logistic model to glean the adjusted odds ratios and associated confidence intervals. Tests were conducted at *α *= .005 using SPSS Version 27.

## Results

### Participant characteristics

Most participants were; males (58.5%), first-year students; (37.2%), had no chronic condition; (89.0%), were non-alcohol consumers (66.5%), non-smoking (88.2%), of normal BMI (64.7%) and sufficiently physically active (82.6) and had experienced an adverse event in the past month (77.4%). The mean perceived barriers and benefits to PA scores were 95 ± 11.4 and 28.6 ± 5.4, respectively. Physical performance 25.9 ± 3.4 and life enhancement 24.7 ± 3.5 were the most cited benefits of exercising as exhibited by the highest mean scores. The most perceived barriers to exercise were a lack of exercise infrastructure of 14.1 ± 3.0 and physical exertion of 7.9 ± 1.6. Last, 33.5% of students are at risk of CMDs while 17.4% of students were classified as having low PA levels. Last, participants had a high self-rated HRQoL evaluation; mean EQ-5 5D VAS 80.5 ± 13.5 (Table 1).

**Table 1:** Participants’ characteristics *N* = 465.

Variable	Attribute	*n* (%)
Gender	Male	193 (41.5)
	Female	272 (58.5)
*Age	Mean (SD)	21.7 ± 1.6
Study year	1^st^	173 (37.2)
	2^nd^	145 (31.2)
	3^rd^	50 (10.8)
	4^th^	97 (20.9)
Adverse events	Yes	360 (77.4)
	No	105 (22.6)
Chronic conditions	Present	51 (11.0)
	Absent	414 (89.0)
Smoking	Yes	55 (11.8)
	No	410 (88.2)
Alcohol use	Yes	156 (33.5)
	No	309 (66.5)
Substance use	Yes	42 (9.0)
	No	423 (91.0)
Financial adequacy	Inadequate	119 (25.6)
	Adequate	346 (74.4)
Food security	Yes	270 (58.1)
	No	195 (41.9)
*Benefits sub-scale mean (SD)	Physical performance	25.9 ± 3.4
	Life enhancement	24.7 ± 3.5
	Psychological outlook	19.0 ± 2.9
	Social interaction	11.5 ± 2.2
	Prevention health	9.6 ± 1.5
	Total Benefits score	95 ± 11.4
*Barriers subscale mean (SD)	Time expenditure	6.8 ± 1.7
	Physical exertion	7.9 ± 1.6
	Exercise infrastructure	14.1 ± 3.0
	Family discouragement	4.1 ± 1.4
	Total barriers score	28.6 ± 5.4
Physical activity levels	Low	81 (17.4)
classification	Moderate	178 (38.3)
(IPAQ categories)	High	206 (44.3)
SQ-8 total score	Normal range ≤5	309 (66.5)
	At risk ≥6	156 (33.5)
*EQ-5D 5L utility score	Mean (SD)	.845 (SD.084)
*EQ-5D 5L VAS score	Mean (SD)	80.5 (SD 13.5)
BMI categories	Underweight	25 (4.9)
	Normal weight	301 (64.7)
	Pre-obesity	89 (19.1)
	Obesity	27 (5.8)

All variables marked with an * are not reported in the n (frequency) format but rather in the mean (SD) format.

### Correlations between study variables

Experience of barriers to exercise was associated with lower HRQoL (*r* = −.168; *p* < .001) and high CMDs (*r* = .224: *p* < .001). Conversely, more significant perceived benefits to exercise were associated with higher HRQoL (*r* = .226; *p* < .001)—See Supplementary Table S1.

### Crude odds ratios

Univariate binary logistic regression shows these variables were associated with increased odds of experiencing barriers to exercise: experiencing an adverse event (OR = 2.21: 95% CI 1.39–3.51), having a chronic condition (OR = 1.91: 95% CI 1.03–3.56), being a current smoker (OR = 2.17, 95% CI 1.17–4.00), inadequate finances (OR = 2.13: 95% CI 1.40–3.23), and food insecurity (OR = 3.38: 95% CI 2.28–5.01) (proxy socio-economic indicator), and poor mental health (OR = 2.7: 95% CI 1.77–4.02). Not taking substances (OR = 1.84: 95% CI.97–3.48) and higher perceived HRQoL (OR = 24.69: 95% CI 2.08–292.51) were associated with increased odds of higher perceived exercise benefits (Table 2).

**Table 2:** Factors associated with barriers and facilitators (unadjusted odds ratios).

Variable	Attribute	Barriers crude odds ratio		Benefits crude odds ratio	
		OR (CI)	*p*-value	OR (CI)	*p*-value
Gender	Male	1		1	
	Female	.97 (.67: 1.41)	0.888	1.25 (.85: 1.80)	0.268
Age		.94 (.84: 1.05)	0.239	.93 (.83: 1.04)	0.194
Study year	1^st^	.85 (.52: 1.40)	0.519	.73 (.44: 1.22)	0.232
	2^nd^	.93 (.55: 1.55)	0.771	.85 (.50: 1.44)	0.542
	3^rd^	.87 (.44: 1.73)	0.693	.31 (.31: 1.23)	0.169
	4^th^	1		1	
Faculty	Medical Faculty	1.01 (.60: 1.70)	0.98	.82 (.49: 1.37)	0.441
	Non-medical faculty	1		1	
Adverse events	Yes	2.21 (1.39: 3.51)	**<.001**	.92 (.59: 1.43)	0.718
	No	1		1	
Chronic conditions	Present	1.91 (1.03: 3.56)	**0**.**041**	.99 (.55: 1.77)	0.963
	Absent	1		1	
Smoking	Yes	2.17 (1.17: 4.00)	**0**.**013**		
	No	1		.99 (.56: 1.75)	0.98
Alcohol use	Yes	1.27 (.86: 1.88)	0.228	1	
	No	1		1.12 (.76: 1.65)	0.583
Substance use	Yes	1.93 (.97: 3.81)	**0**.**059**	1	
	No	1		1.84 (.97: 3.48)	0.063
Financial adequacy	Yes	1		1	
	No	2.13 (1.40: 3.23)	**<**.**001**	0.94 (.62: 1.43)	0.78
Food security	Yes	1		1.05 (.72: 1.52)	0.807
	No	3.38 (2.28: 5.01)	**<**.**001**		
Physical activity	Low	1.42 (.85: 2.37)	0.186	.81 (.48: 1.37)	0.428
Levels/categories	Medium	1.03 (.68: 1.54)	0.903	.97 (.65: 1.45)	0.869
	High	1		1	
SSQ-8 score classification	Normal ≤5	1		.953 (88: 1.03)	0.22
	At risk ≥6	2.7 (1.77; 4.02)	**<**.**0001**	1	
EQ-5D 5L	Utility Score	.009 (.001:.13)	**<.001**	24.69 (2.08:292.51)	**0**.**011**
EQ-5D 5L	VAS score	.98 (.97: 1.00)	**0**.**018**	1.01 (1.00: 1.03)	**0**.**074**
BMI classifications	Underweight	.66 (.21: 2.13)	0.489	2. 75 (.79: 9.55)	0.615
	Normal weight	1.33 (.59: 3.00)	0.495	.94 (.42: 2.10)	0.884
	Pre-obesity	1.99 (.82: 4.82)	0.128	.59 (.25: 1.41)	0.232
	Obesity	1		1	

Values in bold are statistically significant with *p* ≤ 0.05.

### Adjusted odds ratios

After controlling for co-variation and confounding, only food insecurity (AOR 2.51: 95% CI 1.62–3.88) and the risk of CMDs (AOR 0.49: 95% CI 0.32–0.76) were associated with increased odds of experiencing barriers to exercise. Not using substances (AOR = 2.14: 95% CI 1.11–4.14) and a higher self-rated HRQoL (AOR 24.34: 95% CI 1.77–335.13) were associated with increased odds of a high perception of exercise benefits (Table 3).

**Table 3:** Factors associated with barriers and facilitators (adjusted odds ratios).

Domain	Factor	B	S.E.	Wald	df	Sig.	AOR	95% C.I. (lower: upper limit)
Barriers	Adverse event	−0.35	0.26	1.88	1	0.171	0.70	0.42–1.17
	Smoking	−0.51	0.34	2.33	1	0.127	0.60	0.31–1.16
	Risk of CMDs	−0.71	0.22	10.21	1	**0** **.** **001**	0.49	0.32–0.76
	Financial inadequacy	0.37	0.24	2.45	1	0.118	1.45	0.91–2.32
	Food insecurity	0.92	0.22	17.18	1	**<**.**001**	2.51	1.62–3.88
	Constant	1.70	0.42	16.22	1	<.001	5.47	
Benefits	EQ-5D 5L utility score	3.19	1.34	5.69	1	**0**.**017**	24.34	1.77–335.13
	EQ-5D 5L VAS score	0.01	0.01	1.23	1	0.268	1.01	0.99–1.02
	Substances non-intake	0.76	0.34	5.10	1	**0**.**024**	2.14	1.11–4.14
	Constant	−3.71	1.14	10.53	1	0.001	0.02	

Values in bold are statistically significant with *p* ≤ 0.05.

## Discussion

This study evaluated the perceived barriers and facilitators to physical activity in Zimbabwean university students and associated factors. Overall, our study outcomes show that university students were strongly knowledgeable of the benefits of exercising with physical performance, life enhancement and psychological impacts cited as major benefits. Time constraints, physical exertion, and lack of exercise infrastructure were major barriers to exercise. Also, a higher quality of life was associated with an increased perception of benefits. Last, lower socio-economic status and poor mental health were associated with increased barriers to exercise.

Participants scored highly on the benefits subscale, i.e., a mean of 95 ± 11.4. Our results are comparable to studies done on American and Saudi Arabian university students, which yielded mean EBBS benefits subscale scores of 94 ± 11 and 95 ± 10, respectively (27, 44). University students are likely to have high health literacy, contributing to the high perception of the benefits of exercise (14). A high educational level is linked to increased positive health-information-seeking behaviours and high health literacy, which may be invariably linked to favourable perceptions of physical activity's health benefits (14, 45). Also, university students are exposed to various health promotional activities, such as campus-wide health expos and educational talks, which may increase their knowledge of the benefits of exercise (46). At the sub-domain level, university students highly endorsed the positive effects of exercise on physical performance, life enhancement and psychological impacts. Elsewhere, South African physiotherapy students (*N* = 296) highly ranked the physical performance and psychological impacts of exercise (14). This was attributable to the advanced health education in the physiotherapy training curriculum (14). Other studies have shown that preventive health, physical performance and life enhancement are high motivators of exercise engagement among university students (24, 27). For instance, university students are likely to engage in physical activity as a stress-coping mechanism (24, 27).

In this study, university students experienced moderate barriers to exercising, with a mean barriers’ subscale of 28.6 ± 5.4. These results are comparable to studies done on American and Indian university students, which yielded mean EBBS barriers subscales of 28.5 ± 6.7 and 29.5 ± 7, respectively (44, 47). The high comparability suggests that barriers to exercise among university students are universal across socio-economic contexts. At the subscale level, the most perceived barriers to exercising were time constraints, physical exertion, and exercise infrastructure. Most students perceived exercise as taking too much time from studies and family responsibilities. This finding was similarly reported in Spanish (48), Saudi Arabian (49) and German students (50), who reported that they would instead use their free time to prepare for exams or socialising than engage in physical exercise. As noted in a systematic review (51), our outcomes show that physical exertion is a barrier to exercising by students. Exercise can be physically and cognitively demanding, and if not addressed, perpetual fatigue may lead to decreased PA engagement. A South African also showed that time constraints and physical exertion are substantial barriers (38). Also, the lack of exercise facilities was a significant barrier to exercise engagement in this study. This was unsurprising as there are few to no state-subsidized on-campus exercise facilities in Zimbabwe. Elsewhere, 75% of Saudi university students cited the lack of public exercise facilities as a barrier to exercising (52). In contrast, in a study on German students (*N* = 689), only 2.9% perceived the availability of facilities/programs as a barrier to exercise as the university sports program is offered at low to no cost (50).

Most participants were classified as having moderate to high PA levels. These reported levels of PA engagement may be attributed to structural challenges university students face in this context. In a study to document objective PA trajectories in students in the USA (*N* = 805), participants were more active during structured days (i.e., school days); this finding was expected because most students had to walk to or around campus during weekdays similar to the setting in our study (53). Further, a study among Indian university students (*N* = 255) showed that the high to moderate physical activity levels among university students might be due to environmental factors, including lack of access to reliable on-campus transportation. Consequently, students tend to be more physically active when reliant on active transportation, such as cycling or walking to and around campus (54). No association was found between PA levels and barriers or benefits to exercise. Other factors may have influenced physical activity levels in students in this sample.

Unadjusted odds ratios from the univariate analysis suggests that poor mental health and low socio-economic status are salient barriers to PA engagement in university students. Although these unadjusted outcomes are subject to confounding and co-variation biases, our study findings showed a high burden of CMDs in university students. Also, poor mental health was associated with an increased likelihood of perceived barriers to exercise. Poor mental health functioning is related to a lack of energy and motivation for PA engagement, creating a vicious cycle of poor physical and mental health (55, 56). A systematic review by Sheldon et al. (57) reported similar findings: negative exercise perceptions were associated with an increased risk of CMDs in international undergraduates. Also, participants with chronic conditions reported more exercise barriers. Chronic illness is associated with lethargy, bodily pain and fatigue, contributing to reduced physical endurance; thus, participants would perceive more barriers to physical activity engagement (30). Notably, a chronic condition, such as CMDs, acts as a stress inducer, adding to the pre-existing academic stress in students, which may exacerbate exercise-avoidance behaviours (55, 56).

University students of lower socioeconomic status were likelier to experience significant exercise barriers. Studies have shown that reduced physical activity engagement among the low SES population is due to a lack of resources, low motivation, fatigue and lack of energy and financial restrictions (58–61). In our context, students may not afford monthly off-campus gym facilities subscriptions due to the prevailing socio-economic challenges. Lastly, our study also shows that university students with a higher self-assessed HRQoL were likelier to perceive exercise benefits. Other studies have indicated that perceived benefits to physical activity were associated with an increased compulsion to greater physical engagement. Increased PA engagement then leads to reductions in the risk of NCDs, leading to increased HRQoL overall (14, 62, 63).

## Conclusion

Our study shows that university students strongly perceive the benefits of exercise with an associated higher HRQoL. Also, poor mental health, low socio-economic status, chronic illness, a lack of exercise infrastructure, time constraints and physical exertion were significant barriers to exercise in this setting. Zimbabwean universities are encouraged to invest in several initiatives to increase PA in university students. For instance, considerations should be made to promote the use of non-conventional exercise spaces like open spaces with adequate security and lighting, allowing students access at any time. Significantly, health promotion activities, including physical activity counselling and education, may increase awareness of the health benefits of PA, including dispelling the perceived myths and barriers around regular physical activity engagement.

### Study limitations

There are limitations to our study. First, the limited study setting of only one centre does not allow conclusions to be drawn about university students in Zimbabwe. Still, the UZ is the largest tertiary institution in Zimbabwe; this may increase the study’s external validity. Second, physical activity data were measured by a self-reported questionnaire, which may have resulted in some students offering socially desirable responses. The IPAQ has yet to be validated in Zimbabwe; this may have introduced measurement bias in PA ascertainment. Future studies should measure PA engagement objectively rather than only relying on subjective measures, which can be inconsistent and make comparing PA patterns among different samples difficult. Also, there is a need for cross-cultural translation and adaptation of extensively used PA outcome measures with evidence of psychometric robustness. Third, we employed a cross-sectional study design, which does not allow for the inference of causality between the variables. Finally, a consecutive sampling method was used for selecting the study sample; this may have introduced selection bias. Where possible, future studies should endeavour to apply random sampling.

### Study strengths

Our study in-cooperated a large sample size (*n* = 465) exceeding the least required sample size of 388; this increases the study's internal validity. Also, to the best of our knowledge, this study is the first to explore the barriers and benefits among university students in Zimbabwe, thus creating context-based information essential for evidence-based care, which may inform future policy formulation.

## Data Availability

The raw data supporting the conclusions of this article will be made available by the authors, without undue reservation.

## Supplementary material

Supplementary File S1 Shows the items on the Exercise Benefits and Barriers Scale (EBBS). The EBBS is a 43-item questionnaire scored on a four-point Likert scale. Responses range from four (strongly agree) to one (strongly disagree). The benefit and barriers components comprise 29 and 14 items, respectively.

Supplementary File S2 Shows the correlation between exercise barriers, benefits, physical activity, common mental disorders, body mass index, and HRQoL.
