# Supplementary material for: Perceived benefits and barriers to exercise and associated factors among Zimbabwean undergraduate students: a cross-sectional study
Source: Front Sports Act Living. 2024 Aug 7;6:1205914. doi: 10.3389/fspor.2024.1205914 (PMC11335725; doi:10.3389/fspor.2024.1205914)
Supplement: Supplementary File S2 — Shows the correlation between exercise barriers, benefits, physical activity, common mental disorders, body mass index, and HRQoL. [file Table1.docx]

Additional File 1: Exercise Benefits and Barriers Scale (EBBS)

| **Sub scale** | **Domain** | **Question** |
| --- | --- | --- |
| **Benefits** | **Physical performance** | 7. Exercise increases my muscle strength. |
|  |  | 15. Exercising increases my level of physical fitness. |
|  |  | 17. My muscle tone is improved with exercise. |
|  |  | 18. Exercising improves my functioning cardiovascular system. |
|  |  | 22. Exercise increases my stamina |
|  |  | 23. Exercise improves my flexibility. |
|  |  | 31. My physical endurance is improved by exercising. |
|  |  | 43. Exercise improves the way my body looks |
|  | **Life enhancement** | 25. My disposition is improved with exercise. |
|  |  | 26. Exercising helps me sleep better at night. |
|  |  | 29. Exercise helps me decrease fatigue. |
|  |  | 32. Exercising improves my self-concept. |
|  |  | 34. Exercising increases my mental alertness |
|  |  | 35. Exercise allows me to carry out normal activities without becoming tired. |
|  |  | 36. Exercise improves the quality of my work |
|  |  | 41. Exercise improves overall body functioning for me. |
|  | **Psychological outlook** | 1. I enjoy exercise. |
|  |  | 2. Exercise decreases feelings of stress and tension for me. |
|  |  | 3. Exercise improves my mental health. |
|  |  | 8. Exercise gives me a sense of personal accomplishment. |
|  |  | 10. Exercising makes me feel relaxed |
|  |  | 20. I have improved feelings of well-being from exercise. |
|  | **Social interaction** | 11. Exercising lets me have contact with friends and persons I enjoy. |
|  |  | 30. Exercising is a good way for me to meet new people. |
|  |  | 38. Exercise is good entertainment for me. |
|  |  | 39. Exercising increases my acceptance by others. |
|  | **Preventive health** | I will prevent heart attacks by exercising. |
|  |  | 13. Exercising will keep me from having high blood pressure. |
|  |  | 27. I will live longer if I exercise. |
| **Barriers** | **Time expenditure** | 5. Exercising takes too much of my time |
|  |  | 24. Exercise takes too much time from family relationships. |
|  |  | 37. Exercise takes too much time from my family responsibilities. |
|  | **Physical exertion** | 6. Exercise tires me |
|  |  | 19. I am fatigued by exercise. |
|  |  | 40. Exercise is hard work for me. |
|  | **Exercise infrastructure** | 9. Places for me to exercise are too far away. |
|  |  | 12. I am too embarrassed to exercise. |
|  |  | 14. It costs too much to exercise |
|  |  | 16. Exercise facilities do not have convenient schedules for me. |
|  |  | 28. I think people in exercise clothes look funny |
|  |  | 42. There are too few places for me to exercise. |
|  | **Family discouragement** | 21. My spouse (or significant other) does not encourage exercising. |
|  |  | 33. My family members do not encourage me to exercise |

Additional File 2: Correlations between study variables, N=465

|  |  | IPAQ TOTAL Score (METs/week) | Barriers Subscale Total | Benefits Subscale Total | EQ-5D 5L Utility Score | EQ-5D 5L VAS Score | SSQ-8 Total Score | BMI |
| --- | --- | --- | --- | --- | --- | --- | --- | --- |
| Barriers | Pearson Correlation | 0.023 | 1 | -0.033 | -.168^**^ | -.117^*^ | .224^**^ | -0.002 |
| Subscale | Sig. (2-tailed) | 0.614 |  | 0.474 | 0.000 | 0.012 | 0.000 | 0.967 |
| Benefits | Pearson Correlation | 0.009 | -0.033 | 1 | .226^**^ | .140^**^ | -0.069 | 0.069 |
| Subscale | Sig. (2-tailed) | 0.845 | 0.474 |  | 0.000 | 0.002 | 0.139 | 0.143 |

**. Correlation is significant at the 0.01 level (2-tailed). *. Correlation is significant at the 0.05 level (2-tailed).
